# Novel method to remove deeply migrated pancreatic duct stent with caudal end embedded in the pancreas

**DOI:** 10.1055/a-2590-7817

**Published:** 2025-05-19

**Authors:** Lianqiang Song, Fan Cheng, Xiaofei Liu, Yongshuai Liu, Hongmei Qu, Shanming Sun, Qiang Tian

**Affiliations:** 1 117907 Department of Gastroenterology, Weifang Peopleʼs Hospital, Weifang, China

A 63-year-old man underwent a routine endoscopic retrograde cholangiopancreatography (ERCP) for biliary pancreatitis. During stone extraction, a single pigtail 5-Fr plastic pancreatic duct (PD) stent was inserted into the caudal PD of the pancreatic body due to malpractice, and a 7-Fr single pigtail stent was inserted into the PD of the head of the pancreas ( Fig. 1 ). The attempts to retrieve the stent with snares were not successful.

**Fig. 1:**
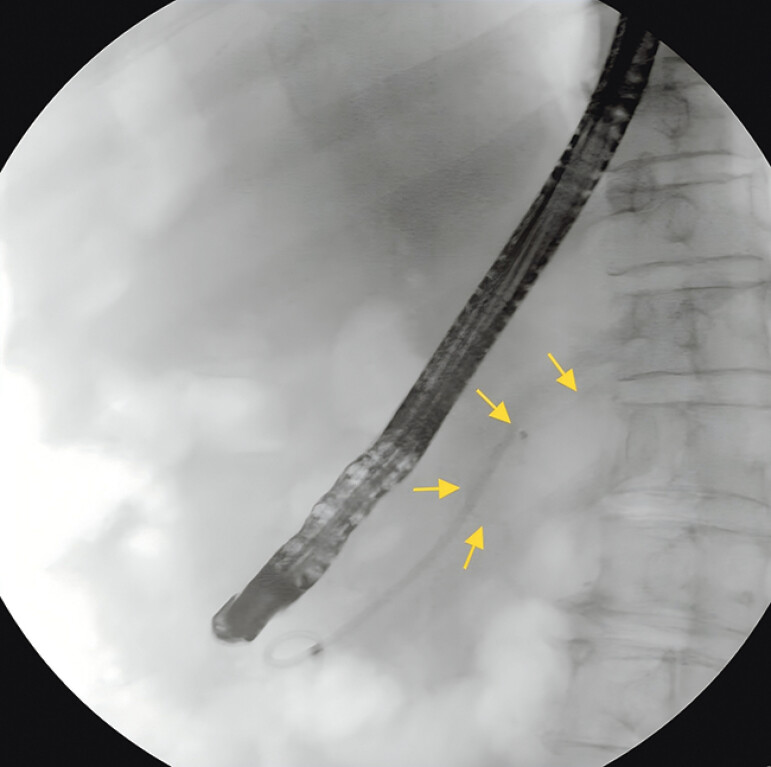
Radiography indicated that the stent was displaced into the pancreatic duct.

ERCP was performed 3 months later, and the attempts to retrieve the stent with various accessories (snares, biopsy forceps, and balloons) were not successful ( Fig. 2 ). We performed PD stent removal with the aid of the eyeMax (Micro-Tech Co Ltd, Nanjing, China) direct visualization system. A mini-basket was placed under direct vision to remove the tail end of the PD stent, which was unsuccessful due to the embedding of the tail end of the stent in the PD ( Fig. 3 ). We tried to use the mini-basket to cross the stent on one side and open the minibasket to place the snares in PD. Under direct vision of eyeMax, we adjusted the position and depth of the snares to cross the stent and then used the minibasket to successfully attach the snares ( Fig. 4 ), and then we successfully took out the PD stent by pulling on the eyeMax, the minibasket, and the snares ( Video 1 ). A 5-Fr single pigtail PD stent was placed to prevent postoperative pancreatitis. The pancreatic duct stent was removed after 2 weeks of follow-up.

**Fig. 2:**
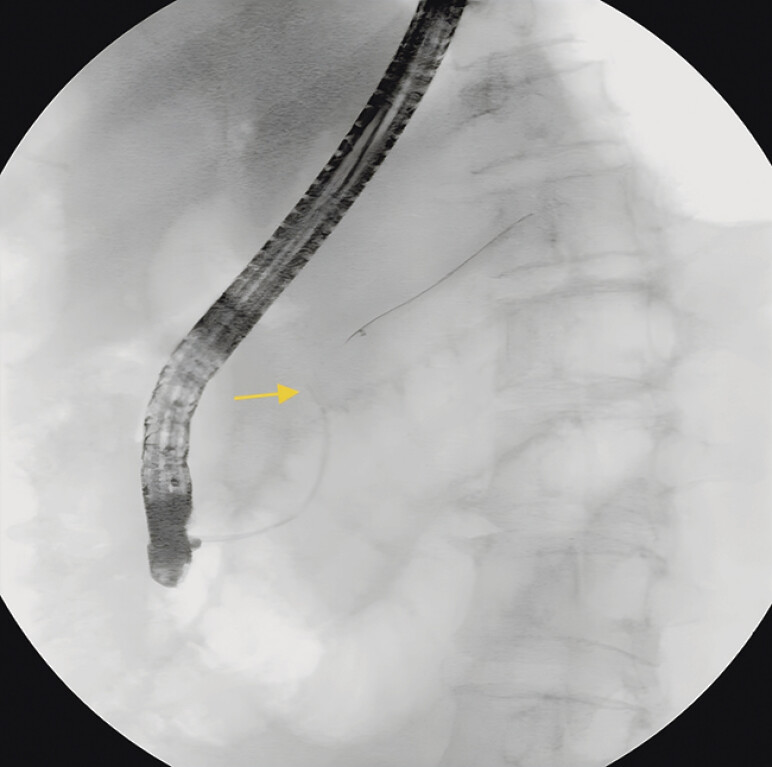
The attempts to retrieve the stent with snares were not successful.

**Fig. 3:**
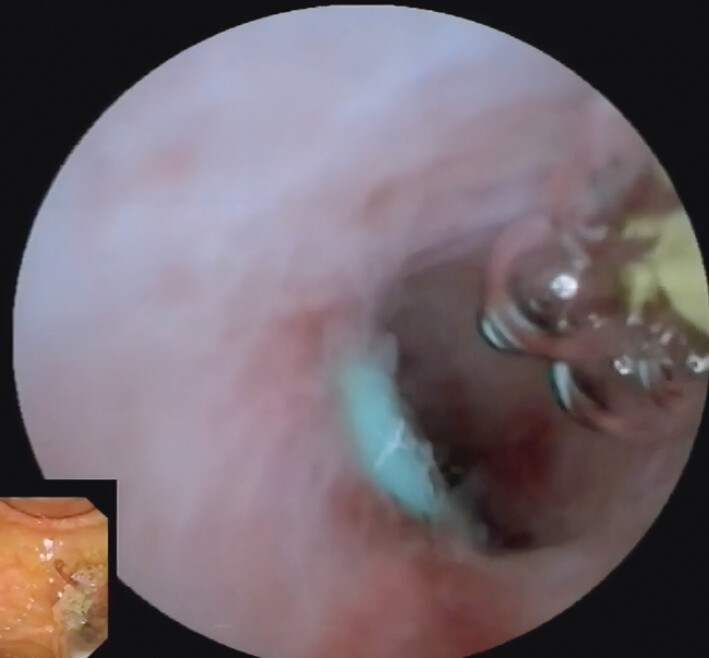
EyeMax showed a stent tail embedded in the pancreatic duct.

**Fig. 4:**
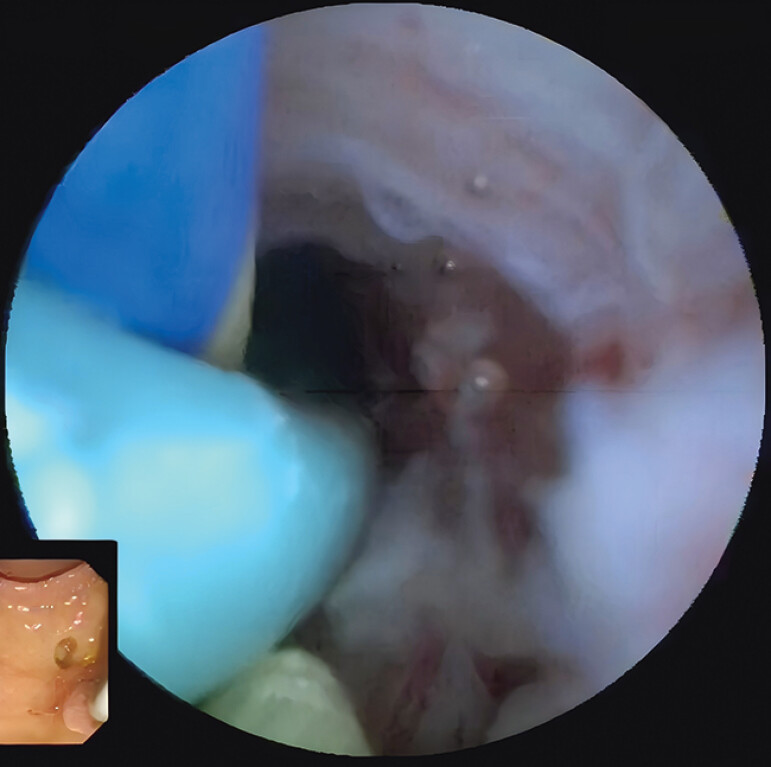
Used the mini-basket to successfully attach the snares.

Novel “closed-loop” method that successfully removes an internally migrated PD stent embedded caudally in the pancreas.Video 1

Many techniques have been described for the removal of proximally displaced PD stents 1 2 . It is more difficult if the stent is located in the tail of the pancreas. We report another novel “closed-loop” method that successfully removes an internally migrated PD stent embedded caudally in the pancreas, providing a new method for the future removal of migrated PD stents.

Endoscopy_UCTN_Code_CPL_1AK_2AD

## Notes

Endoscopy E-Videos https://eref.thieme.de/e-videos
*E-Videos* is an open access online section of the journal *Endoscopy* , reporting on interesting cases and new techniques in gastroenterological endoscopy. All papers include a high-quality video and are published with a Creative Commons CC-BY license. Endoscopy E-Videos qualify for HINARI discounts and waivers and eligibility is automatically checked during the submission process. We grant 100% waivers to articles whose corresponding authors are based in Group A countries and 50% waivers to those who are based in Group B countries as classified by Research4Life (see: https://www.research4life.org/access/eligibility/ ). This section has its own submission website at https://mc.manuscriptcentral.com/e-videos .
